# Origin and spatial population structure of Malagasy native chickens based on mitochondrial DNA

**DOI:** 10.1038/s41598-023-50708-x

**Published:** 2024-01-04

**Authors:** Takahiro Yonezawa, Hideyuki Mannen, Kaho Honma, Megumi Matsunaga, Felix Rakotondraparany, Fanomezana Mihaja Ratsoavina, Jiaqi Wu, Masahide Nishibori, Yoshio Yamamoto

**Affiliations:** 1 Graduate School of Integrated Sciences for Life, Hiroshima University, Higashi-Hiroshima, 739-8528 Japan; 2 Faculty of Agriculture, Tokyo University of Agriculture, 1737 Funako, Atsugi, Kanagawa 243-0034 Japan; 3 Laboratory of Animal Breeding and Genetics, Graduate School of Agricultural Science, Kobe University, Nada, Kobe, 657-8501 Japan; 4 Graduate School of Biosphere Science, Hiroshima University, Higashi-Hiroshima, 739-8528 Japan; 5 Chubu Regional Office, Agriculture and Forestry Bureau, Tottori, 682-0802 Japan; 6 Department of Zoology and Animal Biodiversity, Faculty of Science, University of Antananarivo, BP 906, 101 Antananarivo, Madagascar; 7 Department of Molecular Life Science, Tokai University School of Medicine, 143 Shimo-Kasuya, Isehara, Kanagawa 259-1193 Japan

**Keywords:** Population genetics, Evolutionary genetics

## Abstract

Since Malagasy human culture became established in a multi-layered way by genetic admixture of Austronesian (Indonesia), Bantu (East Africa) and West Asian populations, the Malagasy native livestock should also have originated from these regions. While recent genetic studies revealed that Malagasy native dogs and goats were propagated from Africa, the origin of Malagasy native chickens is still controversial. Here, we conducted a phylogeographic analysis of the native chickens, focusing on the historical relationships among the Indian Ocean rim countries and based on mitochondrial *D-loop* sequences. Although previous work suggested that the rare Haplogroup D occurs with high frequencies in Island Southeast Asia–Pacific, East Africa and Madagascar, the major mitochondrial lineage in Malagasy populations is actually not Haplogroup D but the Sub-haplogroup C2, which is also observed in East Africa, North Africa, India and West Asia. We demonstrate that the Malagasy native chickens were propagated directly from West Asia (including India and North Africa), and not via East Africa. Furthermore, they display clear genetic differentiation within Madagascar, separated into the Highland and Lowland regions as seen in the human genomic landscape on this island. Our findings provide new insights for better understanding the intercommunion of material/non-material cultures within and around Madagascar.

## Introduction

As the Malagasy biota is characterized by species-richness and high endemism, its geographical isolation from other lands as well as the topographical configurations within its huge landmass must have greatly influenced the evolution of its organisms^1,2^, including humans. A recent population genomic study clarified that the Malagasy people were mainly established by separate migrations of Austronesians from Borneo around 2–3000 years ago and of Bantu from East Africa around 1500 years ago, with additional limited paternal contributions from Middle Eastern people^3^. This finding indicates that Madagascar represents an intersection of distinct culture spheres. Pierron et al.^3^ further demonstrated that there are clear spatial genetic structures on this island which have been maintained up to the present: genetic components of Bantu in coastal regions and of Austronesians in the Highlands.

Domestic animals and cultivated plants have left deep impacts on human history^4^. They have played important roles not only as a food resource, but also in socio-cultural aspects. Since they have been propagated mainly by human mediation, it is important to reveal when and from where they have been propagated, and how population structure has been maintained, to better understand the intercommunion of material and non-material cultures. However, little is currently known about the origins and genetic structures of Malagasy native livestock. Recent genetic studies have clarified that Malagasy native dogs^5^ and goats^6,7^ have East/South African origins. Linguistic evidence also suggests that Malagasy terms for these animals (*Amboa* for dogs and *Osy* for goats) were drawn from Swahili or other Coastal Bantu^8^.

As for chickens, Osman et al.^9^ suggested a genetic affinity between East African chickens and Pacific chickens, and proposed that Austronesians mediated chicken propagation to East Africa. On the other hand, Herrera et al.^10^ extensively analyzed Island Southeast Asian (ISEA) and Pacific chickens and compared them with Malagasy chickens that had been characterized by Razafindraibe et al.^11^. They noted the genetic affinity of Malagasy and East African chickens, and further suggested that Malagasy and East African chickens are genetically closer to Indian chickens than to ISEA/Pacific chickens.

However, as linguistic evidence indicates that the origin of the Malagasy term for chickens (*akoho*) cannot be definitely assigned to Bantu or Austronesian^8^, the evolutionary relationships among the native chickens of Madagascar and other regions of the Indian Ocean rim (e.g., West Asia and North Africa) should also be examined to elucidate the origin of Malagasy chickens. In addition, the Malagasy chickens analyzed by Herrera et al.^10^ were limited to two local subpopulations in the Central Highland, and probably do not sufficiently represent the genetic diversity of Malagasy chickens. The evolutionary relationship between Malagasy and East African chickens is therefore still controversial. In addition, little is known about the population structure of chickens within Madagascar. The purpose of this study was to elucidate the origin and phylogeographic structure of Malagasy native chickens by analyzing widely sampled chickens in Madagascar.

## Results and discussion

### Evolutionary relationships between ISEA/Pacific chickens and Malagasy chickens

**Figure 1:**
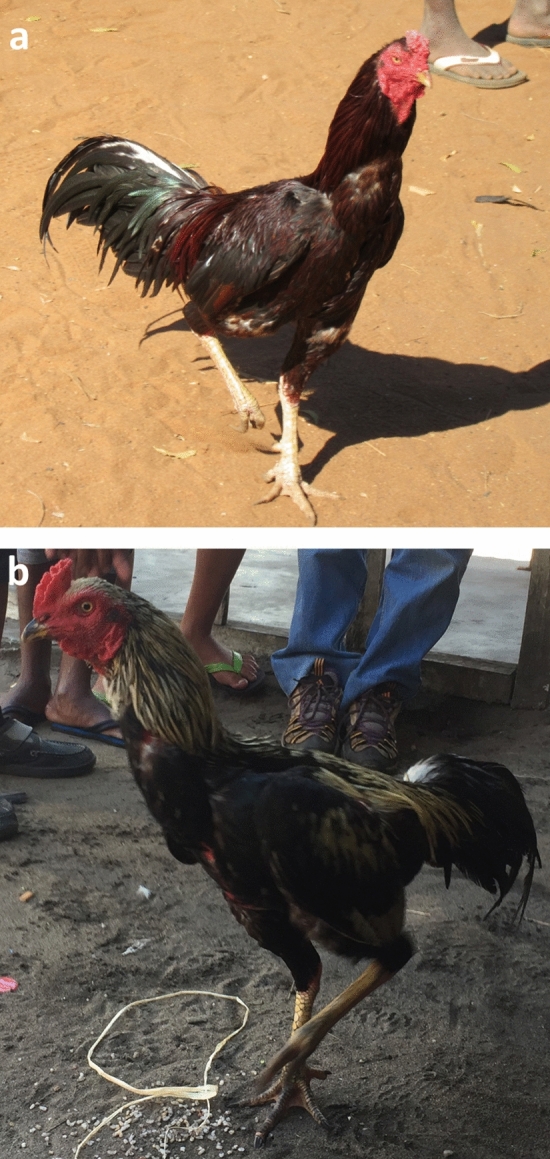
External appearance of Malagasy native chickens. (**a**) Native chicken kept in the local village on the south coast of Madagascar (Beloha, Tulear). (**b**) Native chickens kept on the east coast of Madagascar (Mahanoro, Toamasina). Malagasy native chickens show the standing posture with wide chest that is seen in cockfighting breeds represented by “Malay”.

**Figure 2:**
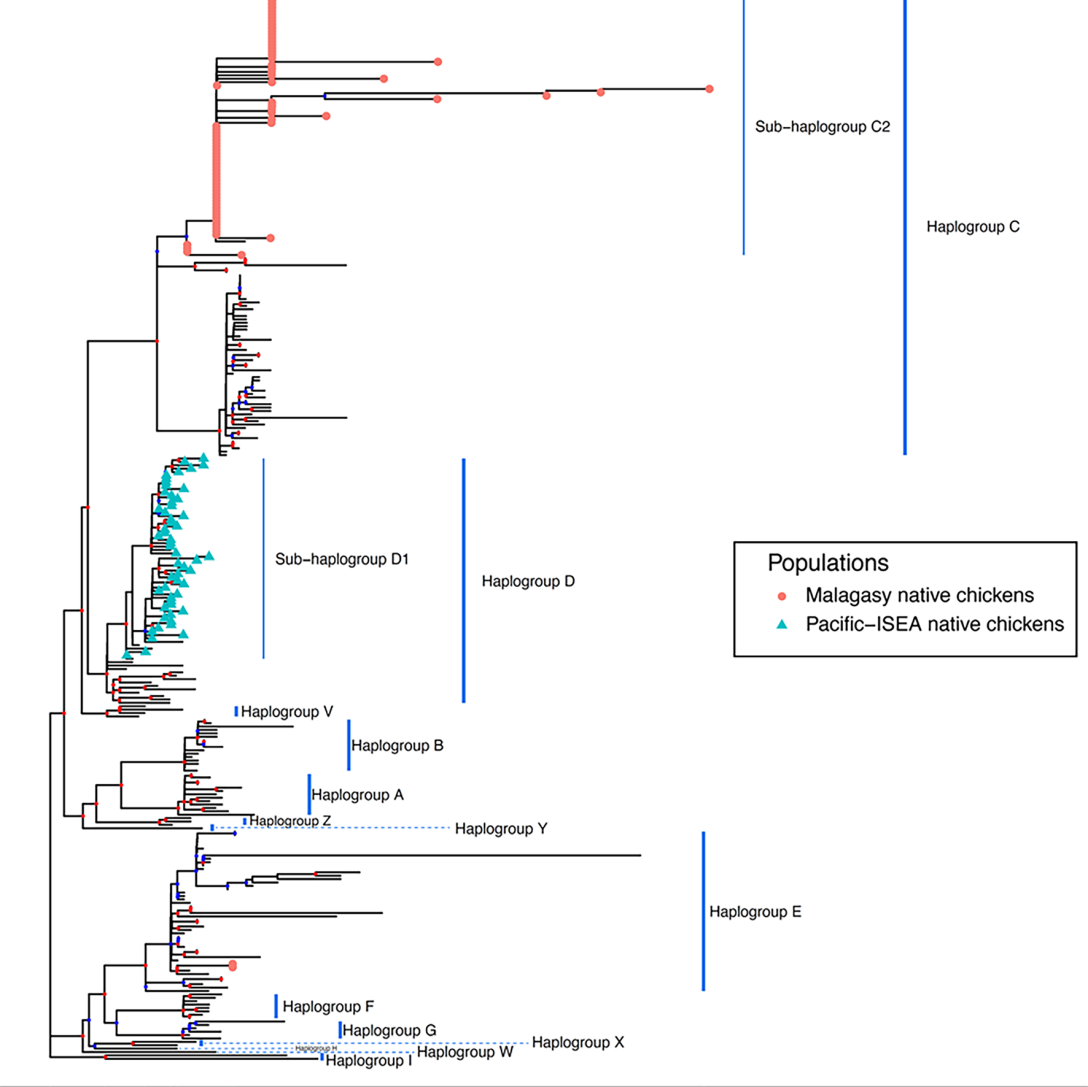
Phylogenetic positions of Malagasy native chickens. The phylogenetic positions of the Malagasy native chickens (red triangles) among domestic chickens and red junglefowls were determined by the ML method. Complete *D-loop* sequences were used for the Malagasy native chickens and mitochondrial genomes were used for other birds. Pacific-ISEA native chickens (turquoise blue triangles) belong to Haplogroup D, whereas the Malagasy native chickens belong to Sub-haplogroup C2 except for two from Fort Dauphin (Haplogroup E). Branch lengths are proportional to the numbers of nucleotide substitutions per site; scale bar, 0.0002 nucleotide substitutions per site. Nodal color indicates the bootstrap probabilities (red ≧ 80%, 80% > blue≧ 50%). Detailed information for each sequence (e.g., accession numbers, individual IDs, bootstrap probabilities) as well as the nodal support values are shown in Supplementary Figure S1.

Interestingly, all the village chickens we observed in Madagascar show a standing posture with a wide chest reminiscent of fighting breeds such as "Malay" (Fig. 1). Hereafter, we refer to this morphotype of chicken as the Malay type. An ML tree based on the super-matrix of the complete mt genomes of 230 domestic chickens/red junglefowls as well as the complete *D-loop* sequences of 78 Malagasy native chickens is shown in Fig. 2. Regarding this super-matrix alignment, since the *D-loop* sequences of Malagasy native chickens were aligned together with the 230 mt genome data, the non-*D-loop* regions of Malagasy native chickens contain gaps only. When the ML tree was reconstructed, these gap sites were treated as missing data. Accordingly, the backbone structure of the ML tree was mainly reconstructed by the mt genome sequences information, and the subtrees of *D-loop* sequences were placed on this backbone structure. Our previous work^12^ demonstrated this super-matrix approach drastically improved the resolutions of the phylogenetic inference among the mitochondrial haplotypes of chickens. Therefore, it is expected that our ML tree (Fig. 2, Supplementary Figure S1) shows the phylogenetic relationships between the ISEA/Pacific chickens and Malagasy chickens more clearly. All the mt genome sequences from ISEA-Pacific native chickens were situated as Sub-haplogroup D1 within Haplogroup D^13^. On the other hand, all Malagasy chickens examined in this study belonged to Sub-haplogroup C2 except for two from Fort Dauphin. These two birds belonged to Haplogroup E.

In view of the small number of ISEA/Pacific chickens (53 birds) involved in this ML analysis, the *D-loop* sequences of the ISEA/Pacific chickens (1012 birds) reported by Herrera et al.^10^ and Matsunaga et al.^14^ were further added to the basic alignment data. These 1065 (53 mitogenome + 1012 *D-loop* sequence) ISEA/Pacific chickens include the population (46 birds) from Borneo, where the first Malagasy migrant people originated^3^. The ML tree based on 1373 domestic chickens/red junglefowls (230 complete mt genome and 1143 *D-loop* sequences) was inferred as shown in Supplementary Figure S2. Accession numbers or individual IDs in these alignment data are included in this figure. Besides Haplogroup D, some of the ISEA/Pacific chickens belonged to Haplogroup A, Haplogroup B, Sub-haplogroup C1, Haplogroup E, Haplogroup I, and several unknown haplogroups. However, none of the ISEA/Pacific chickens belonged to Sub-haplogroup C2.

As for Haplogroup E, seen in both the ISEA/Pacific chickens and the Malagasy chickens, a BLAST search showed that the two Malagasy chickens of Haplogroup E sampled in Fort Dauphin have a novel haplotype (Hap_21: Table S1). This haplotype is very closely related to haplotype (LC586655), with only one nucleotide difference (identity = 1229/1230 bp). The haplotype (LC586655) is ubiquitously distributed in Eurasia (Japan, China (Henan), Bangladesh, Iran, and Russia (Leningrad)), but does not occur in the ISEA/Pacific regions. Since Fort Dauphin has been an important port city since the 1500 s, gene introgression from a Eurasian foreign breed is possible. These findings strongly suggest that Malagasy chickens are not descended from ISEA/Pacific chickens.

### Origin of Malagasy native chickens

**Figure 3:**
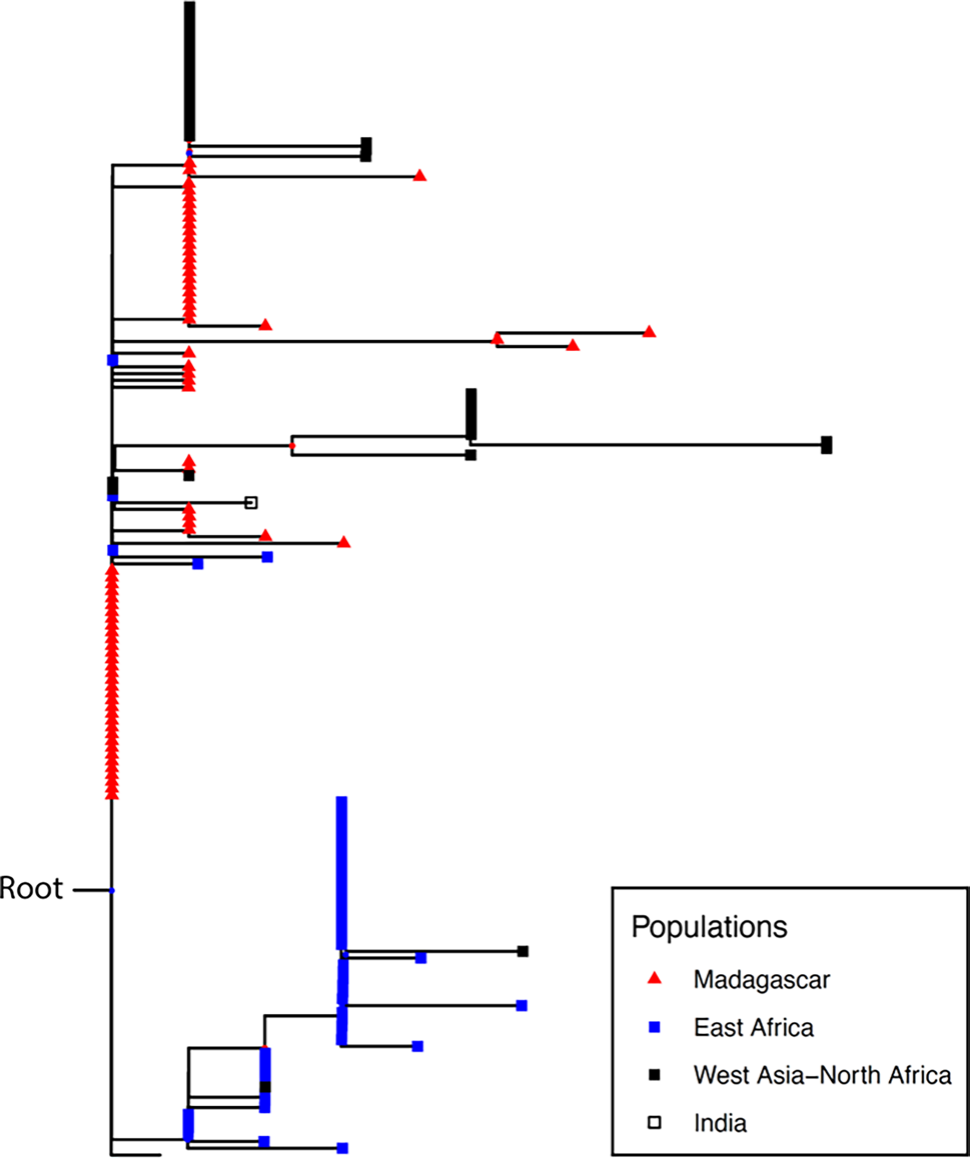
Phylogenetic relationships within Sub-haplogroup C2. The ML tree is based on the *D-loop* sequences of native chickens from the Indian Ocean Rim (Madagascar, East Africa, India, and West Asia: 1320 individuals) as well as the complete mt genomes of 230 domestic chickens/red junglefowls. A magnified view of Sub-haplogroup C2 is shown. The geographic regions of the native chickens are distinguished by the colors of the symbols (red: Madagascar; black: East Africa; blue: West Asia–North Africa; and white: India). Nodal color indicates the bootstrap probabilities (red ≧ 80%, 80% > blue≧ 50%). Branch lengths are proportional to the numbers of nucleotide substitutions per site (scale bar, 0.0002 nucleotide substitutions per site). The whole ML tree including sequence information (e.g., accession numbers, individual IDs, bootstrap probabilities) is shown in Supplementary Figure S3.

Where, then, do the Malagasy native chickens originate from? To elucidate this issue, an ML tree was inferred based on the *D-loop* sequences of chickens from the Indian Ocean rim (Madagascar, East Africa, North Africa, West Asia, and India). The *D-loop* sequences of African, West Asian, and Indian chickens from previous studies (e.g.,^15,16^) were retrieved from NCBI, and aligned together with the basic alignment data. This data set consists of 1554 chickens/red junglefowls (1324 *D-loop* and 230 complete mt genome sequences). Again, the complete mt genomes of 230 domestic chickens/red junglefowls were also used for constructing the backbone structure of the ML tree (Supplementary Figure S3). Accession numbers or individual IDs in the alignment data are provided in this figure. A magnified view of the ML tree focusing on Sub-haplogroup C2 is shown in Fig. 3. Although the reason is unclear, Malagasy 11 from Manja was nested within the Sub-haplogroup D1 only in this analysis. Excluding this point, as consistent with other analyses (Supplementary Figures S1 and S2), all Malagasy native chickens, except for two from Fort Dauphin, belong to Sub-haplogroup C2. In addition, some East African, North African, Indian, and West Asian native chickens also belonged to Sub-haplogroup C2. Hereafter, we include North African and Indian populations in “West Asia”. Although East African native chickens were thought to have a large portion of Haplogroup D^17^, it is often difficult to distinguish Haplogroup C and Haplogroup D in partial *D-loop* sequence comparisons^18^. The present study has demonstrated that one of the major haplogroups in Malagasy as well as East/North African and West Asian populations is actually not Haplogroup D but Sub-haplogroup C2. Overall, our phylogenetic analysis does not support the hypothesis that Austronesians propagated chickens to East Africa^9^.

**Figure 4:**
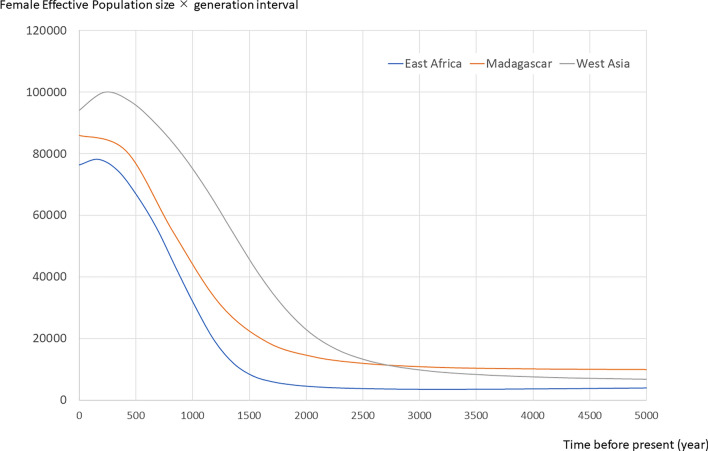
Bayesian Skyline Plot of native chickens from Madagascar, East Africa, and West Asia. Demographic fluctuations of Malagasy (orange line), East African (blue line), and West Asian (gray line) populations were estimated by the Bayesian Skyline Plot. The Y axis indicates the medians of the posterior distributions for the female effective population sizes and the X axis indicates the time before present in years. A mutation rate of 3.13 × 10^–7^ mutations/site/year as well as a generation interval of one year were assumed. West Asian native chickens include Indian and North African individuals.

Regarding the phylogenetic relationships within Sub-haplogroup C2, Malagasy native chickens were mainly located at basal positions, while East African native chickens were at derived positions. This finding calls into question the proposal that Malagasy native chickens originated from East Africa^10^. Indeed, concerning Sub-haplogroup C2, the nucleotide diversity (π) and haplotype diversity (H) of the complete *D-loop* sequences in Malagasy native chickens (π = 0.00141, H = 0.734) are higher than those of East African chickens (π = 0.00096, H = 0.590). Since mt nucleotide diversity is the product of mutation rate and effective female population size, if the Malagasy population is a descendant of an East African population, nucleotide diversity should be lower in Madagascar. Furthermore, we inferred the past population dynamics of Malagasy, East African, and West Asian populations based on Sub-haplogroup C2 by constructing a Bayesian Skyline Plot (Fig. 4). Because Sub-haplogroup C2 was observed in only one individual from India, we included India as part of the West Asian population as mentioned before. The population size of Malagasy native chickens has been larger than that of East African native chickens through history. On the other hand, the West Asian population has generally been larger than the Malagasy population.

**Figure 5:**
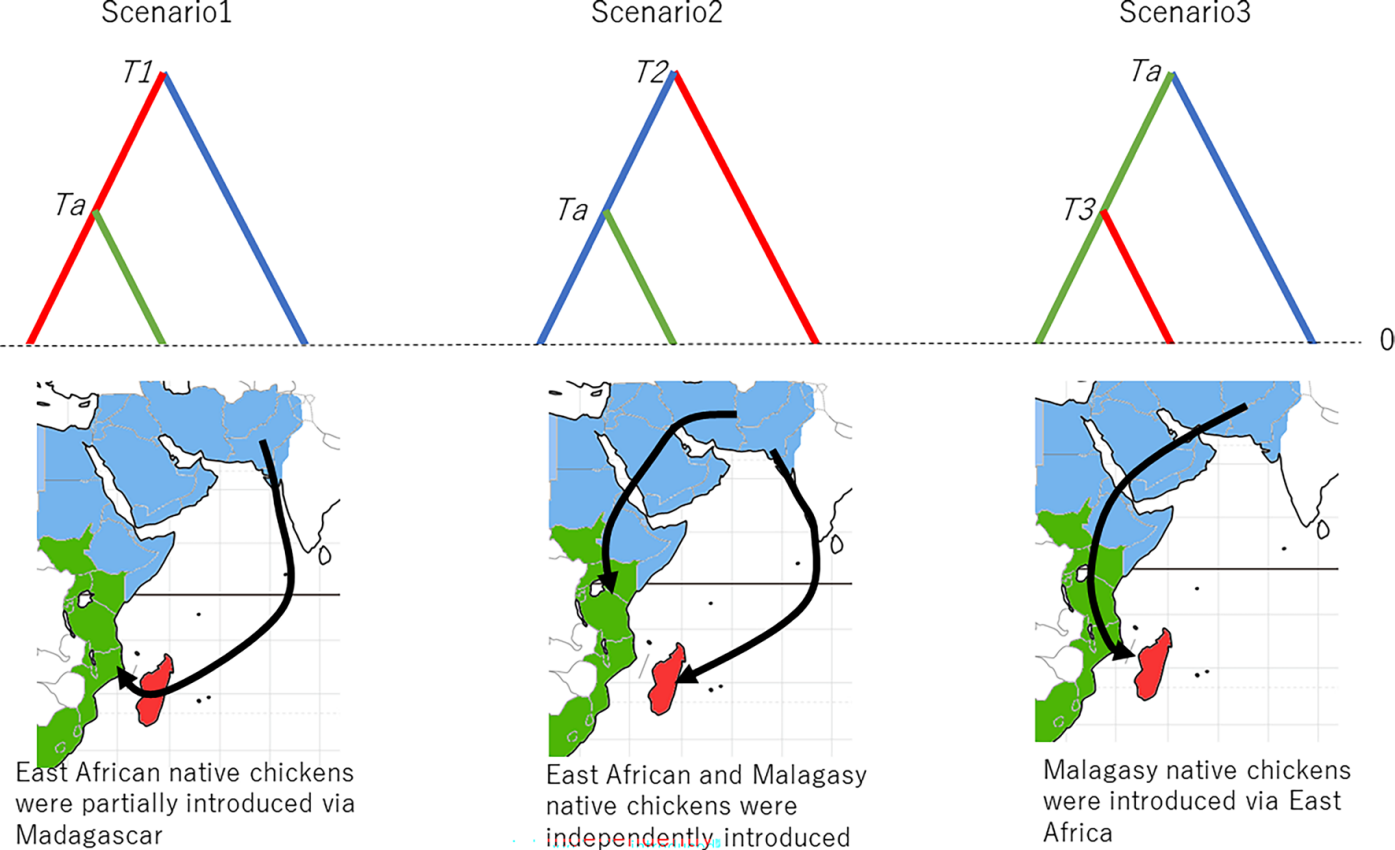
Evolutionary scenarios on the origin of Malagasy native chickens used for ABC. Scenario1: East African native chickens were partially introduced from West Asia via Madagascar. Scenario2: East African and Malagasy native chickens were independently introduced from West Asia. Scenario3: Malagasy native chickens were introduced from West Asia via East Africa. In these scenarios, the West Asian populations include Indian and North African individuals. Upper, schematic views of scenarios indicated by the trees; lower, geographical implications shown on maps. The colors of the branches in the trees and geographical regions on the maps are consistent (red: Madagascar; green: East Africa; and blue: West Asia). “*Ta*” is the time when East African chickens originated; we assumed this to be 800–1200 years ago. “*T*1” and “*T*2” are the possible times when Malagasy chickens were derived from West Asia. The range of *T*1 is 800–2000 years ago (*T*1 > *Ta*), and *T*2 is 700–2000 years ago. “*T*3” is a possible time when Malagasy chickens were derived from East Africa. The range of *T*3 is 700–1200 years ago (*Ta* > *T*3).

From these findings, the following two evolutionary scenarios are possible: the first is that East African native chickens were propagated from West Asia via Madagascar (Scenario1), and the second is that Malagasy and East African native chickens were independently propagated from West Asia (Scenario2). Together with a third scenario, which is the East African origin hypothesis of Malagasy native chickens (Scenario3), we compared these scenarios using the framework of ABC (Fig. 5).

Recent zooarchaeological studies^19,20^ suggest that chickens were propagated to East Africa through Indian Ocean trading networks, possibly much earlier than the introductions via the north of the continent. Since reliable archaeological evidence of East African chickens can be traced back to approximately 800 to 1200 AD^19,21^, we assumed the prior distribution of the time (*Ta*) when East African chickens were derived from Madagascar (Scenario1) or from West Asia (Scenario2 and Scenario3) was ~ 800–1200 years ago.

In Scenario1, the time (*T*1) when the Malagasy chickens were derived from the West Asian chickens should be older than *Ta* (> 800 years ago). We also assumed that *T*1 is younger than 2000 years ago because the oldest description of the early Indian Ocean trading networks in “The Periplus of the Erythraean Sea” from the early first century AD possibly includes Madagascar as one of the African port centers along the coast of East Africa^19^.

As for Scenario2, because this scenario assumes independent origins for the East African and Malagasy chickens, the orders of the times when the East African chickens derived from the West Asia chickens (*Ta*) and the Malagasy chickens were derived from the West Asia chickens (*T*2) were not fixed; however, *T*2 was assumed to be younger than 2000 years ago (see above), and older than 700 years ago because the oldest archaeological evidence of the Malagasy chickens is in the Eleventh–thirteenth centuries AD, as reported by Prendergast et al.^20^.

In Scenario3, the Malagasy chickens are assumed to have originated in East Africa. Accordingly, the time when the Malagasy chickens descended from the East African chickens (*T*3) should be younger than *Ta* (< 1200 years ago), but older than 700 years ago as mentioned above.

PP (posterior probability) values for Scenario1, Scenario2, and Scenario3 were 0.15 (95% CI (confidence interval): 0.11–0.18), 0.72 (95% CI: 0.67–0.77), and 0.13 (95% CI: 0.10–0.17), respectively. This suggests that Malagasy native chickens were directly propagated from West Asia. In Scenario2, the propagation time of Malagasy chickens (*T*2) was estimated to be ~ 1680 years before present (95% highest posterior density: 1010–1980 years before present).

### Spatial population structures of Malagasy native chickens

**Figure 6:**
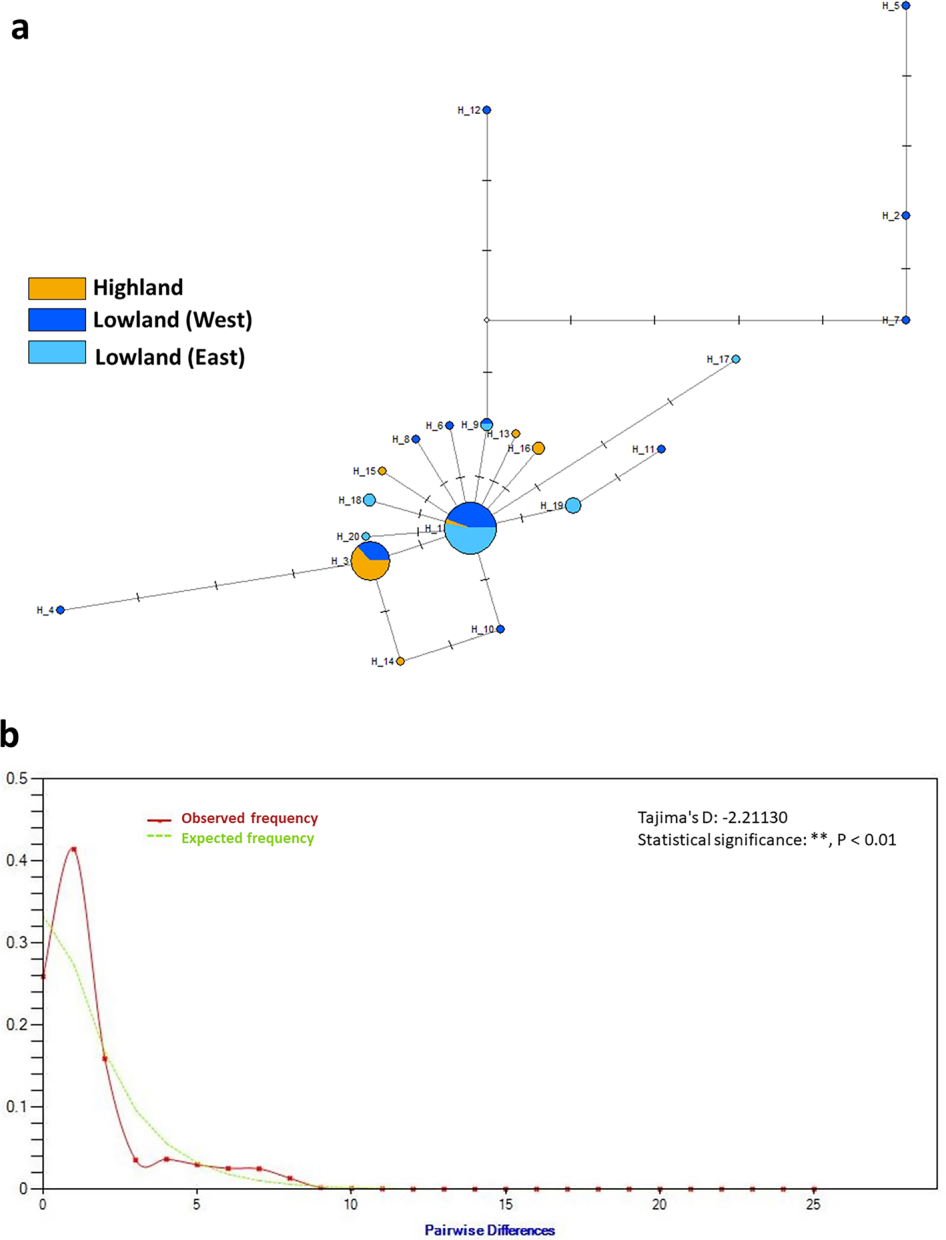
Evolutionary relationships and diversity of the haplotypes in Malagasy native chickens. These analyses are based on the haplotypes of Sub-haplogroup C2. (**a**) MJ network of the haplotypes in Malagasy native chickens. Sizes of the circles are proportional to the haplotype frequencies, and locality information is shown by pie-chart colors (Highland: orange; western Lowland: blue; and eastern Lowland: light blue). Each short bar on the edges represents a nucleotide mutation. (**b**) Mismatch distribution of haplotypes in Malagasy native chickens. Y axis and X axes indicate the frequency and number, respectively, of different nucleotide sites in pairwise comparisons. The red and green curves indicate the observed and expected frequency, respectively. The observed frequency shows unimodal distribution, suggesting recent population expansion, and the significantly negative value of Tajima’s D is concordant with this result.

Although the geographic structures of human beings in Madagascar are well studied from the linguistic and genomic points of view^3,22^, those of domestic animals are little known. Therefore, the spatial population structures of Malagasy native chickens were further investigated in this study. The MJ network of Sub-haplogroup C2 is shown in Fig. 6A. It displays a partial star-like structure, in which minor haplotypes are simultaneously derived from the major haplotype (MAD08). Tajima’s D of Malagasy native chickens is  − 2.20527, and significantly lower than zero (*P* < 0.01). This finding suggests that Malagasy native chickens experienced recent population expansion. The mismatch distribution of Malagasy native chickens is shown in Fig. 6B. τ(= 2μ*t*, where μ is the mutation rate per sequence and *t* is the time of population expansion) was estimated to be 0.248. Alexander et al.^23^ estimated the mt mutation rate of chicken as 3.13 × 10^–7^ mutations/site/year, which corresponds to 3.85 × 10^4^ mutations/sequence/year. Accordingly, *t* is ~ 320 years ago.

**Figure 7:**
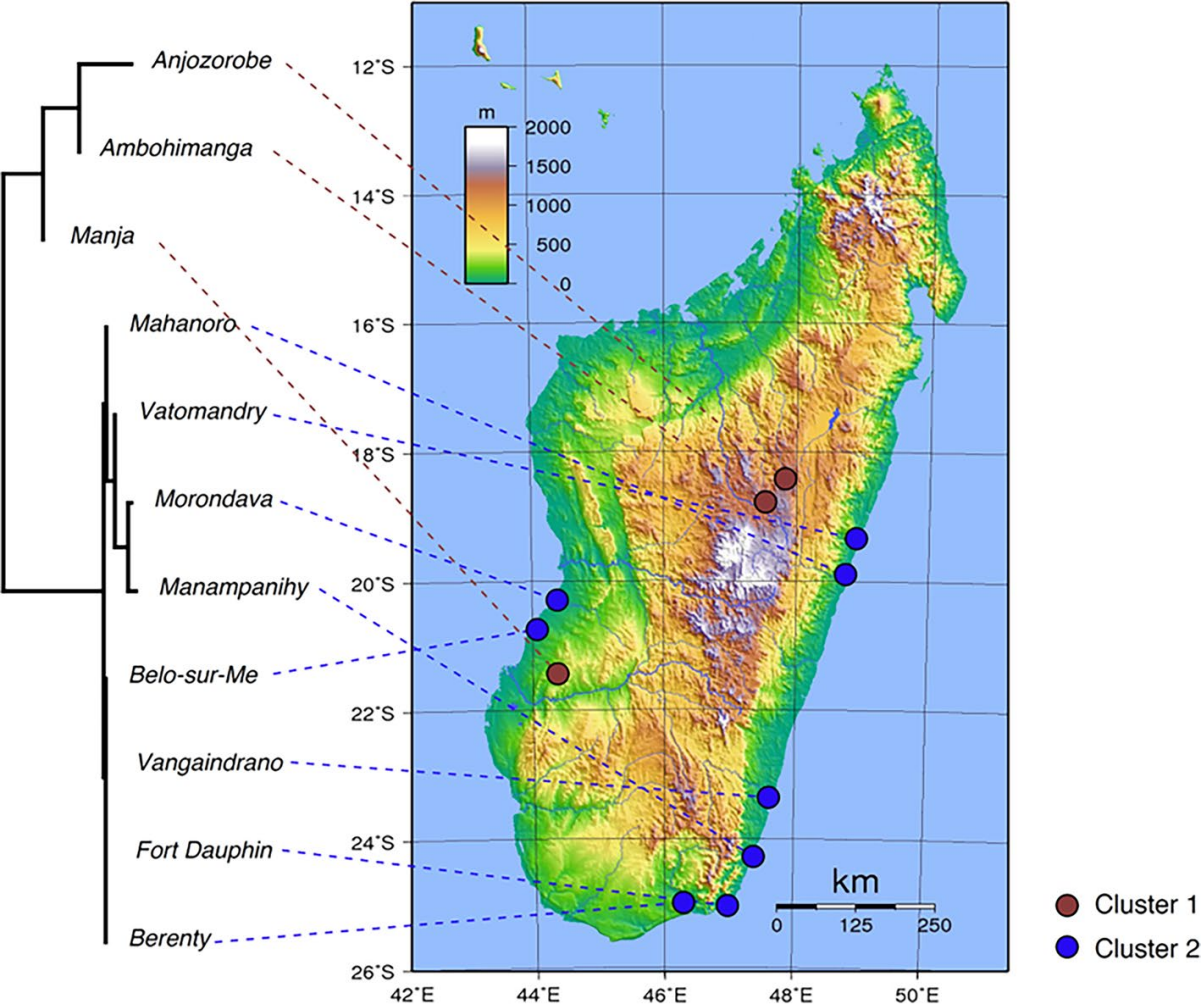
Geographic population structure of Malagasy native chickens. Left: Phylogenetic relationships among the local subpopulations of Malagasy native chickens. The branch lengths are proportional to the net genetic distances among subpopulations (scale bar indicate 0.00005 nucleotide substitutions per site). The root position was determined by the mid-point rooting method with the FigTree program ver. 1.4.4. (https://github.com/rambaut/figtree/releases). Right: The result of the SAMOVA (K = 2). Both results suggest Malagasy population can be separated into the Cluster 1 consists of two highland populations (Ambohimanga and Anjozorobe) + Manja and the Cluster 2 consists of lowland populations (excluding Manja). The topographic map of Madagascar (https://commons.wikimedia.org/wiki/File:Madagascar_Topography.png) is Public Domain.

Phylogenetic relationships among local subpopulations were estimated. The Fort Dauphin subpopulation was separated from the others by a very long branch, probably because, as mentioned above, these two chickens belong to Haplogroup E (Fig. 2). Therefore, after excluding these two, a phylogenetic tree among local subpopulations was inferred (Fig. 7). Chickens from the Lowland (< 1000 m) and Highland (> 1000 m) regions were clearly separated. Within the Lowland group, subpopulations from the east and west coasts were highly intermingled, and no clear genetic structure was found. Although the traffic infrastructure of the Malagasy east coast is undeveloped (Supplementary Figure S4), this finding suggests that chickens have frequently been interchanged within Lowland localities despite the inconvenient transportation. Chickens from the Highland [Ambohimanga (1445 m) and Anjozorobe (1270 m)] form a clade, which further forms larger clade with the Lowland Manja subpopulation (west coast). This finding suggests that Highland chickens were derived from Lowland animals, and propagated from the region around Manja. Indeed, the elevation of Manja (240 m) is higher than those of the sampling locations of Lowland (< 30 m) in this work. SAMOVA based on chickens of Haplogroup C2 shows an essentially consistent result. When Malagasy native chickens were separated into two groups (K = 2), Ambohimanga, Anjozorobe, and Manja subpopulations were separated from the others (Fig. 7).

### New scenario on the origin and history of Malagasy native chickens

In addition to Austronesians and Bantu, Arabs from West Asia genetically contributed to establish Malagasy people^3^. Madagascar had played an important role as a transoceanic trading hub connecting ports of the Indian Ocean ever since the first arrival of Arabs, thought to be between the seventh and ninth centuries AD for trading^24^ or even the early first century AD as mentioned above. Our genetic analysis suggests that Malagasy native chickens originated from West Asia (Scenario1 + Scenario2: PP = 0.87). A recent genomic study also suggested that house mice (*Mus musculus*) were propagated from West Asia^25^. A phylogeographic study of the house shrew (*Suncus murinus*–*S*. *montanus* species complex) based on mitochondrial *cytochrome b* sequences also indicated genetic affinity between the Malagasy–Comoro and the West Asian (Yemen) populations^26^. Therefore, it would not be surprising that chickens were propagated to Madagascar by Arabs through Indian Ocean trading together with house mice and house shrews. Since we included North African countries (e.g., Ethiopia) and India as West Asian population in this analysis, the accurate propagation route of the Malagasy native chickens is still unresolved. Accordingly, a full oceanic route directly from the coastal regions of Arabian Sea (or Bay of Bengal) and a partial land route via North Africa are evenly possible.

East African native chickens appear to have been independently introduced from West Asia, probably by a land route (Scenario2) or perhaps by a partially oceanic route via Madagascar (Scenario1). Indeed, in contrast to the Malagasy native chickens that are phenotypically fixed as the Malay type, there are no fighting cock breeds in East Africa except for the Kuchi breed in Tanzania (e.g.,^27^). According to Mushi et al.^28^, the maternal lineage of this breed is Sub-haplogroup C1, which is abundant in East Asia^17,29^, suggesting that the Kuchi breed is not related to Malagasy native chickens. MIGRATE analysis also suggests that there is almost no gene flow between Malagasy and East African native chickens (Table S2).

As for the spatial genetic structure of chickens within Madagascar, our findings clearly indicated that there is genetic differentiation between Highland and Lowland regions. This tendency is consistent with human genetic structure^3^. On the other hand, Serva et al.^22^ proposed that Malagasy dialects can be separated into North, East, and West regions. This suggests that translocation of chickens within this island was mediated by clan migration rather than by cultural transmission. However, this is not a general tendency for livestock: genetic differentiation between Highland and Lowland regions cannot be found in native dogs (Supplementary Figure S5).

The net genetic distance between Highland and Lowland chickens is 2.85 × 10^−4^/site. If the mutation rate proposed by Alexander et al.^23^ is applied, chickens were translocated to Highland region ~ 455 years ago. As mentioned above, the population expansion of Malagasy native chickens occurred ~ 320 years ago. The defensive wall of Ambohimanga Rova (Supplementary Figure S6), situated in the Malagasy Central Highland region, was constructed intermittently from the early eighteenth century to the 1830s by the Merina Kingdom. About 16 million egg whites were purportedly used for the whitewash covering the interior and exterior walls^30^. Our results indicate that there were already sufficiently large numbers of chickens in the Malagasy Highland region to support such a huge construction operation (see Supplementary text).

Moreover, this study has also provided a new insight into a specific feature of Malagasy native chickens: their morphotype is mostly fixed as the Malay type (Fig. 1, Table S1). Although the founder effect or strong artificial selection within island are possible, a clear explanation for why a morphotype typically seen in fighting breeds became fixed even in free-ranging chickens of local farming villages is elusive. Future research based on population genomic data of Malagasy native chickens should shed light on this enigmatic issue.

## Material and methods

### Sample collection, DNA isolation and sequencing

This study is reported in accordance with ARRIVE guidelines (https://arriveguidelines.org). This study was approved by the Department of Zoology and Animal Biodiversity, Faculty of Science, University of Antananarivo, under the attestations the 278/ZBA/16/APR and 234/ZBA/17/ZR, as well as the Hiroshima University Animal Research Committee. All procedures were performed according to their Ethical Guidelines for Academic Research. Whole blood samples were collected from 78 native chickens randomly selected from 11 locations in Madagascar (Table S1). Blood samples, which were collected from the wing veins by skilled persons in a carefully controlled way for minimizing the pain and fear of chickens with the agreement of animal keepers, were stored at 4 °C until DNA extraction. Genomic DNA was extracted from whole blood using the standard phenol/chloroform method^31^. PCR amplification and DNA sequencing of the complete *D-loop* region was conducted as described by Osman and Nishibori^32^. Nucleotide sequences were deposited in the DNA Data Bank of Japan (https://www.ddbj.nig.ac.jp/index.html) under the accession numbers LC706637–LC706714.

### Phylogenetic and population genetic analysis of the population structures of Malagasy native chickens

The complete *D-loop* sequences of Malagasy native chickens were automatically aligned by the MAFFT program ver. 7^33^. Haplotype diversity (H), Tajima’s D^34^ and mismatch distributions were estimated by the DNASP program ver. 6.12.3^35^. The MJ (median joining) network^36^ was constructed by the NETWORK program ver. 10 (https://www.fluxus-engineering.com/sharenet.htm). Phylogenetic relationships among local subpopulations were estimated by the NJ (neighbor joining) method^37^ based on the net-distances without correction of the multiple substitutions using MEGA ver. 7^38^. If the net-distance showed a negative value, we treated is as zero. The nucleotide diversity (π) was also estimated by the MEGA7. Spatial genetic structures were evaluated by the SAMOVA program ver. 2^39^.

### Phylogenetic and population genetic analysis of the origin of Malagasy native chickens

Seventy-eight complete *D-loop* sequences of Malagasy native chickens were automatically aligned by MAFFT ver. 7 together with 230 complete mt (mitochondrial) genome sequences (e.g.,^29^). Hereafter, we call this data set the basic alignment data. Accession numbers or individual IDs in these basic alignment data are shown in Supplementary Figure S1. A phylogenetic tree was inferred by the ML (maximum likelihood) method using the IQ tree program ver. 2^40^ with the optimized molecular substitution model selected by the ModelFinder (Kalyaanamoorthy et al.^41^) using the Bayesian Information Criterion. Gap sites were treated as missing data for this ML analysis.

Subsequently, after eliminating sites with at which > 70% of individuals have missing or ambiguous bases using an in-house Perl program, population genetic analyses were conducted. The nucleotide diversity (π) and haplotype diversity (H) were estimated by DNASP. The past population dynamics were estimated by Bayesian Skyline Plot^42^ using the BEAST program ver.1.10.4^43^.

Migration rates among populations were estimated by the MIGRATE program ver. 3^44^. Evolutionary scenarios on the geographic origin of Malagasy chickens were evaluated by ABC (approximate Bayesian computations) with the DIYABC program ver. 2.1.0^45^. The reference mt genome sequences of Sub-haplogroup C1^17^ were used as outgroups. We conducted simulations 3 million times with the HKY + I + Γ model under the three evolutionary scenarios discussed later.

### Ethical approval

This study was approved by the Department of Zoology and Animal Biodiversity, Faculty of Science, University of Antananarivo, under the attestations the 278/ZBA/16/APR and 234/ZBA/17/ZR, as well as the Hiroshima University Animal Research Committee. All procedures were performed according to their Ethical Guidelines for Academic Research.

### Supplementary Information

Supplementary Information.

## Data Availability

Nucleotide sequences were deposited in DDBJ (DNA Data Bank of Japan; https://www.ddbj.nig.ac.jp/index.html) under the accession numbers LC706637–LC706714.
