# Supplementary material for: Origin and spatial population structure of Malagasy native chickens based on mitochondrial DNA
Source: Sci Rep. 2024 Jan 4;14:569. doi: 10.1038/s41598-023-50708-x (PMC10766636; doi:10.1038/s41598-023-50708-x)
Supplement: Supplementary file 1 — Supplementary Information. [file 41598_2023_50708_MOESM1_ESM.pdf]

Supplementary text

The Bayesian Skyline Plot (Figure 4) estimated in this study indicates that the effective female population size × generation interval had already reached approximately 80,000 by the 1830s, when the defensive walls of Ambohimanga Rova were completed. Although female domestic chickens sexually mature at 180 days on average, we took account of the low productivity of native chickens in the 19th century, and assumed that the generation interval was one year. Accordingly, the effective female population size at that time was about 80,000. Because the effective population size is usually smaller than the census population size, this is the minimal estimate of the population size.

According to Ursule et al. [1], the average egg laying rate of modern Malagasy native chickens on the east coast is 13.2% per day, which corresponds to ~48 eggs per chicken per year. If we assume that the Malagasy native hens in the early 19th century had a similar egg laying rate, they could supply at least ~3.8 million eggs per year.

The defensive walls of Ambohimanga Rova were intermittently constructed/reconstructed from the early 18th century to the 1830s and 16 million eggs are purported to have been used for whitewash (Supplementary Figure S6). If the construction of the defensive walls took ~10–20 years in total, ~0.8–1.6 million eggs were required per year. Because not only chicken eggs, but also duck and goose eggs were used for whitewash, fewer chicken eggs than this total would have been needed. Our minimal estimate of the number of eggs available is thus sufficient for the requirements of this enterprise.

[1] Ursule S.R., Isabelle H.H., Nirina R.R., Conscient Z., Aldiel B., Andry A., and Jules R.A. (2020) Socio-economic Situation of Poultry Farmer and the Local Chicken Production System of the East-Coast of Madagascar. *Universal Journal of Agricultural Research*, 8: 185 – 201. DOI: 10.13189/ujar.2020.080601

Table S1: Sample information

No.	Subpopulation	Geographic cordinate*	Sex	Morphotype	Haplogroup	Haplotype**
Malagasy1	Manja	44.3291, -21.4247	male	Malay	C2	Hap_1
Malagasy2			female	Malay	C2	Hap_1
Malagasy3			female	Malay	C2	Hap_2
Malagasy4			female	Malay	C2	Hap_3
Malagasy5			female	Malay	C2	Hap_3
Malagasy6			male	Malay	C2	Hap_1
Malagasy7			female	Malay	C2	Hap_3
Malagasy8			female	Malay	C2	Hap_3
Malagasy9			male	Malay	C2	Hap_3
Malagasy10			female	Malay	C2	Hap_1
Malagasy11			female	Malay	C2	Hap_4
Malagasy12			female	Malay	C2	Hap_3
Malagasy13			female	Malay	C2	Hap_1
Malagasy14			male	Malay	C2	Hap_3
Malagasy15			female	Malay	C2	Hap_5
Malagasy16	Belo sur Mer	44.0076, -20.7364	male	Malay	C2	Hap_1
Malagasy17			female	Malay	C2	Hap_1
Malagasy18			female	Malay	C2	Hap_1
Malagasy19			male	Malay	C2	Hap_6
Malagasy20			female	Malay	C2	Hap_1
Malagasy21			male	Malay	C2	Hap_1
Malagasy22			male	Malay	C2	Hap_1
Malagasy23	Morondava	44.3185, -20.2788	male	Malay	C2	Hap_1
Malagasy24			male	Malay	C2	Hap_1
Malagasy25			female	Malay	C2	Hap_1
Malagasy26			male	Malay	C2	Hap_7
Malagasy27			female	Malay	C2	Hap_1
Malagasy28			female	Malay	C2	Hap_8
Malagasy29			male	Malay	C2	Hap_9
Malagasy30			female	Malay	C2	Hap_10
Malagasy31			male	Malay	C2	Hap_11
Malagasy32			male	Malay	C2	Hap_12
Malagasy33	Ambohimanga	47.5596, -18.7570	female	Malay	C2	Hap_1
Malagasy34			male	Malay	C2	Hap_3
Malagasy35			female	Malay	C2	Hap_13
Malagasy36			male	Malay	C2	Hap_3
Malagasy37			male	Malay	C2	Hap_14
Malagasy38	Anjozorobe	47.8705, -18.3973	male	Malay	C2	Hap_3
Malagasy39			female	Malay	C2	Hap_3
Malagasy40			female	Malay	C2	Hap_3
Malagasy41			female	Malay	C2	Hap_3
Malagasy42			male	Malay	C2	Hap_3
Malagasy43			male	Malay	C2	Hap_3
Malagasy44			female	Malay	C2	Hap_15
Malagasy45			female	Malay	C2	Hap_3
Malagasy46			female	Malay	C2	Hap_16
Malagasy47			male	Malay	C2	Hap_16
Malagasy48			female	Malay	C2	Hap_3
Malagasy49			male	Malay	C2	Hap_3
Malagasy50			female	Malay	C2	Hap_3
Malagasy51	Berenty	46.2842, -24.9821	female	Malay	C2	Hap_1
Malagasy52			female	Malay	C2	Hap_1
Malagasy53			female	Malay	C2	Hap_1
Malagasy54	Fort Dauphin (Tolagnaro)	46.9662, -25.0295	female	Malay	C2	Hap_1
Malagasy55			female	Malay	C2	Hap_1
Malagasy56			female	Malay	E	NA
Malagasy57			female	Malay	E	NA
Malagasy58	Mahatalaky***	47.0833, -24.7833	male	Malay	C2	Hap_17
Malagasy59	Manampanihy	47.3667, -24.2667	famale	Malay	C2	Hap_18
Malagasy60			female	Malay	C2	Hap_18
Malagasy61			female	Malay	C2	Hap_19
Malagasy62			male	Malay	C2	Hap_20
Malagasy63			male	Malay	C2	Hap_9
Malagasy64	Vangaindrano	47.6029, -23.3468	male	Malay	C2	Hap_1
Malagasy65			female	Malay	C2	Hap_1
Malagasy66			female	Malay	C2	Hap_1
Malagasy67			female	Malay	C2	Hap_1
Malagasy68			female	Malay	C2	Hap_1
Malagasy69	Vatomandry	48.9722, -19.3235	female	Malay	C2	Hap_19
Malagasy70			female	Malay	C2	Hap_1
Malagasy71			male	Malay	C2	Hap_1
Malagasy72			male	Malay	C2	Hap_19
Malagasy73			male	Malay	C2	Hap_1
Malagasy74	Mahanoro	48.8032, -19.8812	male	Malay	C2	Hap_1
Malagasy75			male	Malay	C2	Hap_1
Malagasy76			female	Malay	C2	Hap_1
Malagasy77			female	Malay	C2	Hap_1
Malagasy78			female	Malay	C2	Hap_1

*Geographic cordinates in the east longitude and south latitude used for SAMOVA2 (Figure 7B)

**Haplotype information in the MJ network (Figure 6A)

***This subpopulation was merged with Fort Dauphin subpopulation because of small sample size

Manja, Belo sur Mer, and Morondava were classified as Western Lowland Madagascar.

Berenty, Fort Dauphin, Mahatalaky, Manampanihy, Vangaindrano, Vatomandry, and Mahanoro were classified as Eastern Lowland Madagascar.

Ambohimanga and Anjozorobe were classified Highland Madagascar.

Table S2: Migration rates among East Africa, West Asia, and Madagascar

		To		
		East Africa	West Asia	Madagascar
From	East Africa	**0.00397**	*5.7*	*0.3*
	West Asia	*199.0*	**0.00463**	*2.3*
	Madagascar	*0.3*	*0.3*	**0.00857**

The mode of the posterior distributions are shown.

The diagonal numbers indicate the θ (=2Nfμ) of each populations, where Nf is the female effective population sizes and μ is mutation rate.

Supplementary Figure S1

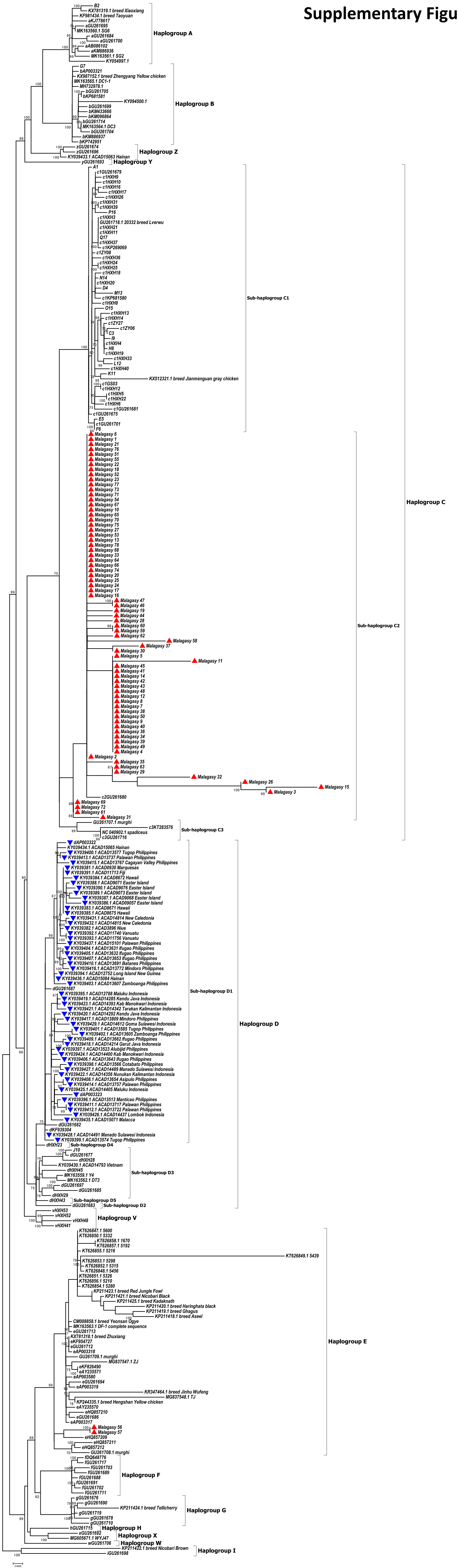

Supplementary Figure S2

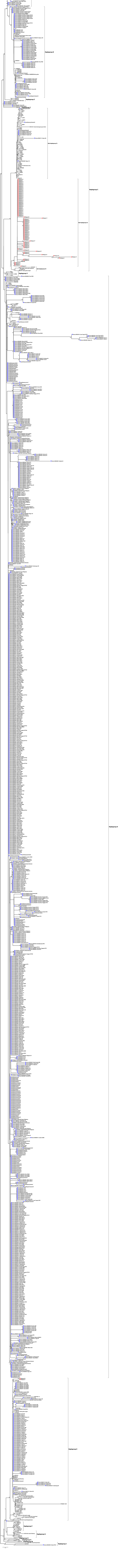

Supplementary Figure S3

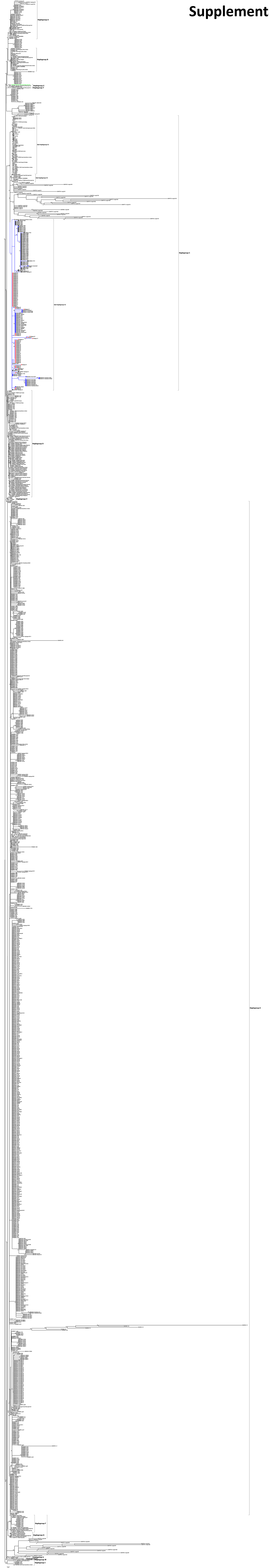

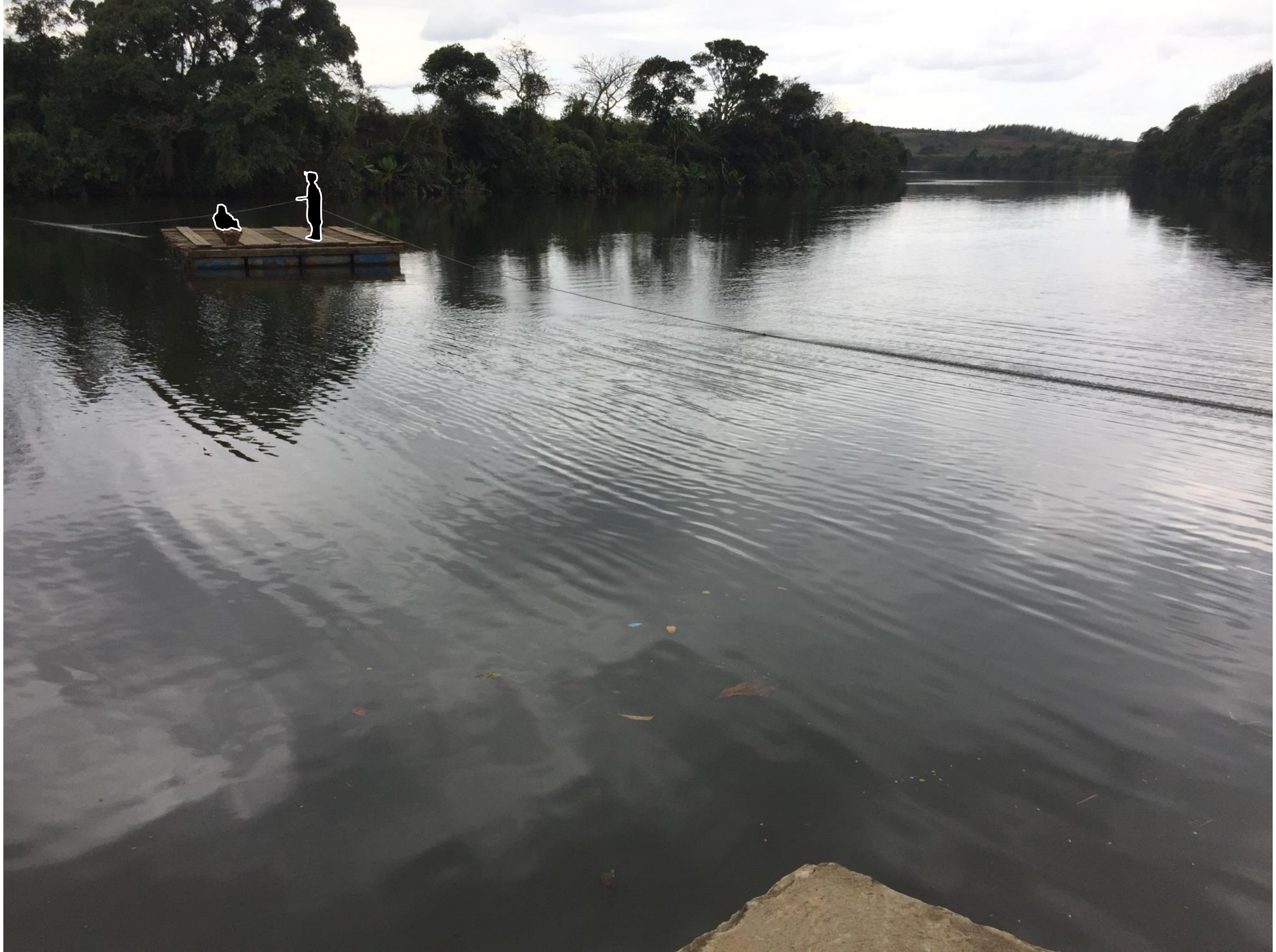

Supplementary Figure S5

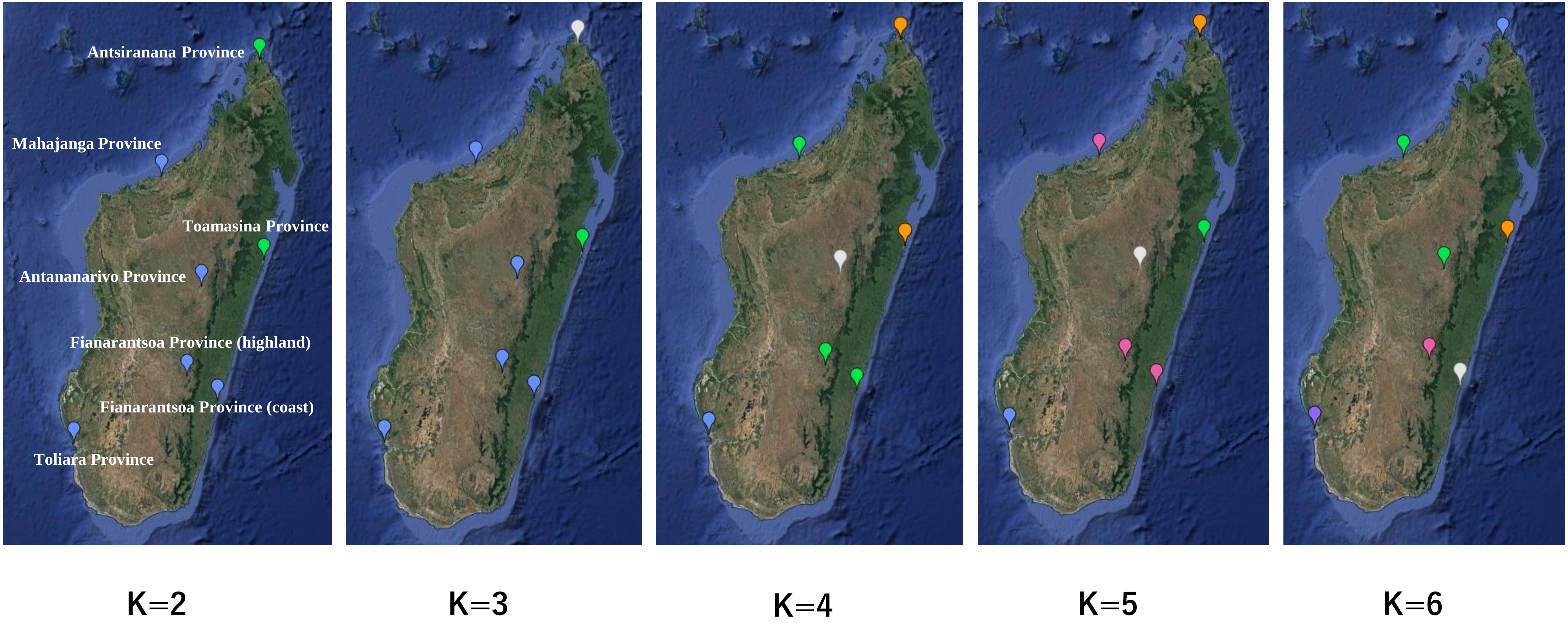

Supplementary Figure S6

(A)

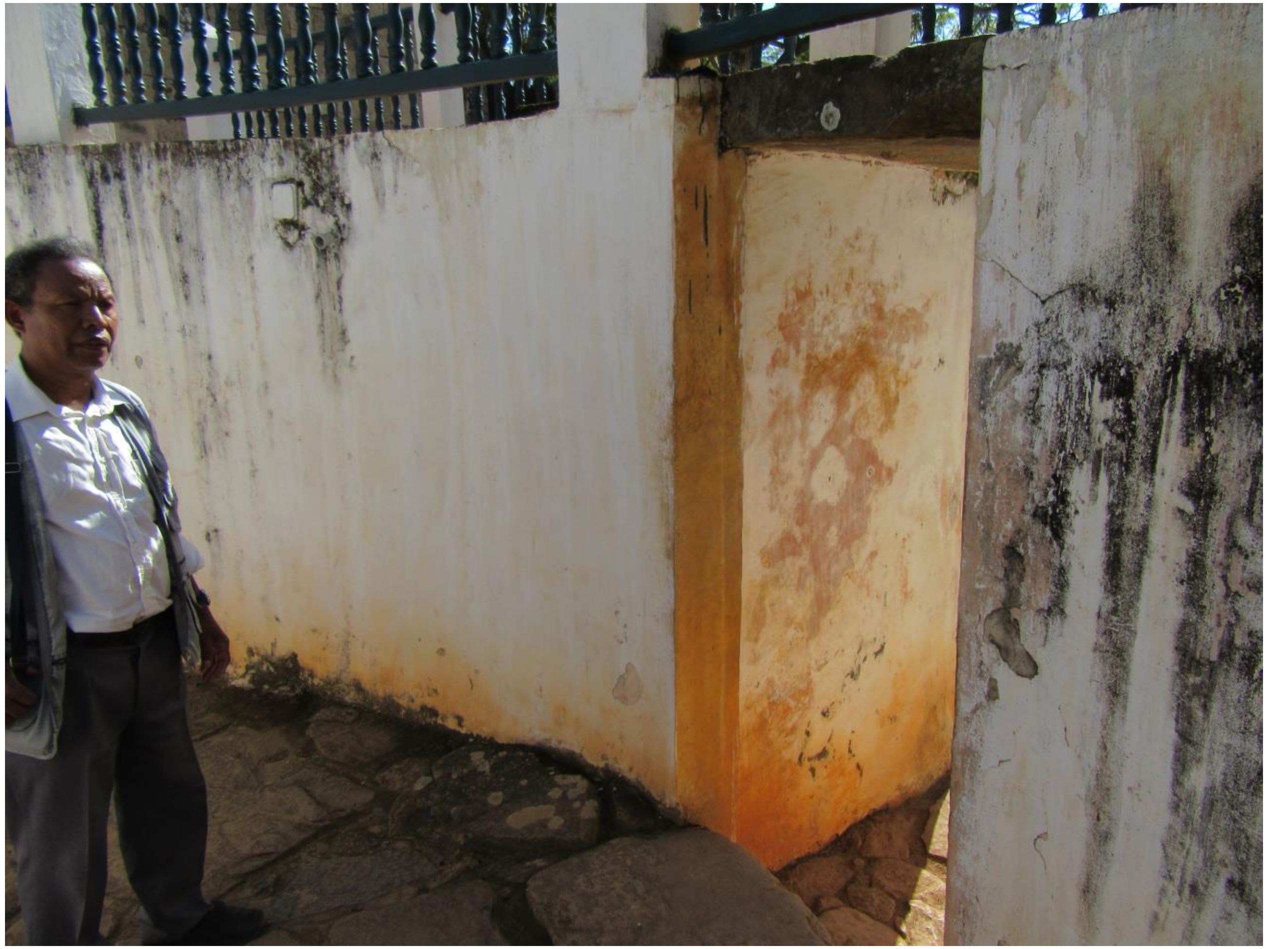

(B)

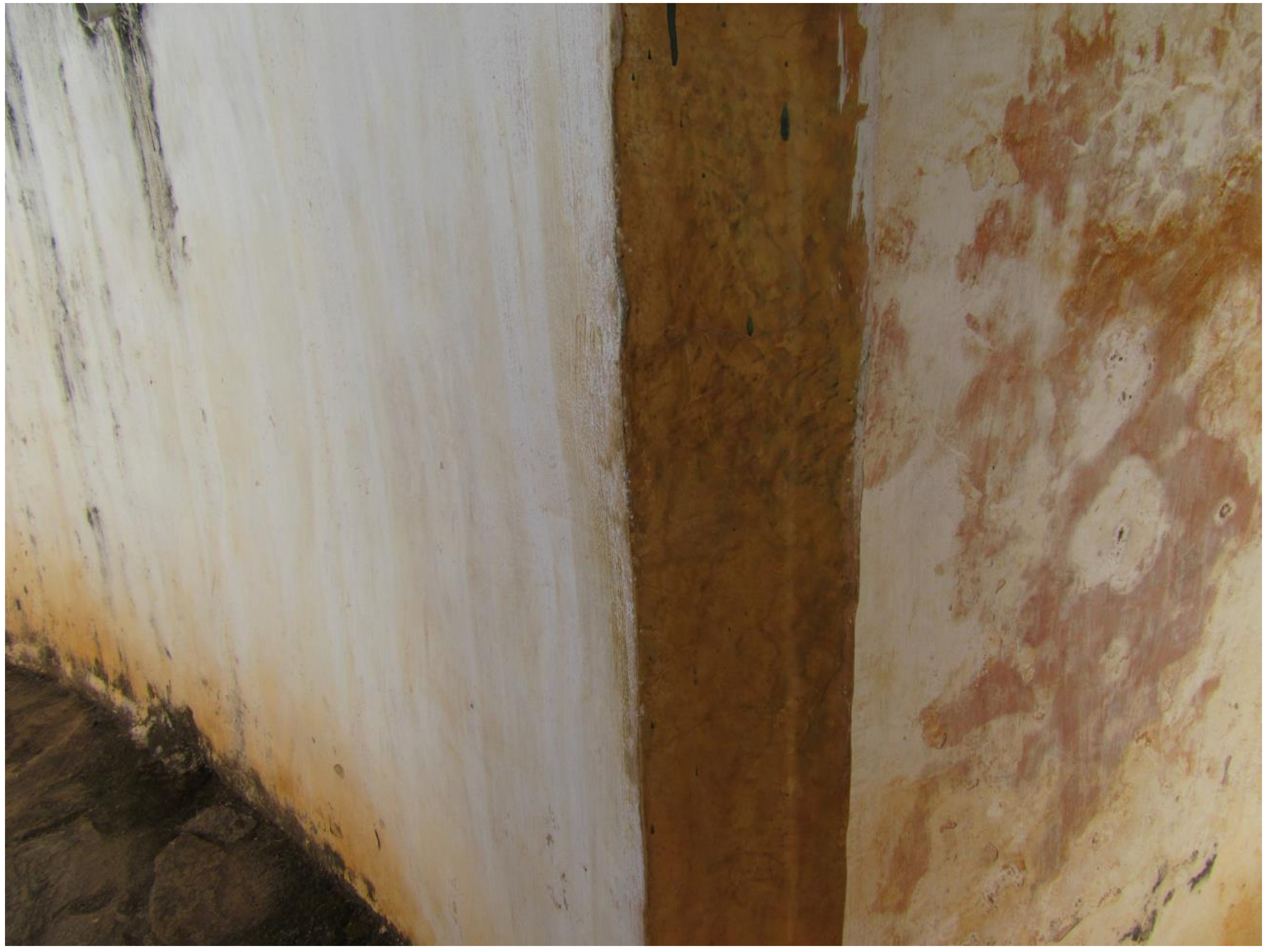

Supplementary figure captions

Supplementary Figure S1: ML tree based on 308 mt DNA sequences of chickens/red junglefowls (230 mt genomes and 78 D-loop sequences). The TN93+F+I+Γ model selected by the BIC was used as the nucleotide substitution model. Nodal numbers indicate the BP (bootstrap probability) with 1,000, replications (only BP values > 70% are shown). Branch lengths are proportional to the numbers of nucleotide substitutions. Red triangles indicate the Malagasy native chickens and blue triangles indicate the ISEA/Pacific native chickens.

Supplementary Figure S2: ML tree based on 1,373 mt DNA sequences of chickens/red junglefowls (230 mt genomes and 1,143 D-loop sequences). The TN93+F+I+Γ model selected by the BIC was used as the nucleotide substitution model. Nodal numbers indicate the BP with 1,000 replications (only BP values > 70% are shown). Branch lengths are proportional to the numbers of nucleotide substitutions. Red closed and blue triangles indicate the Malagasy and ISEA/Pacific native chickens, respectively.

Supplementary Figure S3: ML tree based on 1,554 mt DNA sequences of chickens/red junglefowls (230 mt genomes and 1,324 D-loop sequences). The TIM2+F+I+Γ model selected by the BIC was used as the nucleotide substitution model. Nodal numbers indicate the BP with 1,000 replications (only BP values > 70% are shown). Branch lengths are proportional to the numbers of nucleotide substitutions. In Sub-haplogroup C2, whose branches are colored blue, the Malagasy native chickens are indicated by red triangles, the East African native chickens by closed black squares, the West Asian–North African native chickens by blue squares, and the Indian native chickens by open black squares.

Supplementary Figure S4: A ferry boat in the east coast of Madagascar (Anosy

Region). There are limited numbers of bridges over the rivers in the east coast of Madagascar, and car transportation across the rivers is mainly by ferry boat. These ferry boats are often propelled by human power. Two boat rowers were silhouetted for anonymization.

Supplementary Figure S5: Geographic population structure of Malagasy native dogs. The results of SAMOVA (K = 2–6) are shown. The mitochondrial D-loop sequences of Malagasy native dogs (n = 98) reported by Ardalan et al. (2015) were used for this analysis. The dogs were first subdivided into seven subpopulations (Antananarivo Province (Highland area), part of Fianarantsoa Province (Highland area), part of Fianarantsoa Province (Lowland areas), Antsiranana Province (Lowland areas), Mahajanga Province (Lowland areas), Toamasina Province (Lowland areas), and Toliara Province (Lowland areas)) and clustering by SAMOVA was then conducted.

Supplementary Figure S6: The whitewash of the defensive walls of Ambohimanga Rova. This part of the wall was constructed in 1787, and the whitewash made from the egg white of chickens, ducks, and geese, as well as seashells, chalk, and sands, covering the wall protected it from the erosion. (A) A part of the defensive wall. The person in this photo is one of authors (F. R.). (B) Magnified view of the wall.
